# Trends in cardiovascular risk factors in diabetic patients in comparison to general population in Iran: findings from National Surveys 2007–2016

**DOI:** 10.1038/s41598-020-68640-9

**Published:** 2020-07-16

**Authors:** Hamid Malekzadeh, Mojtaba Lotfaliany, Afshin Ostovar, Farzad Hadaegh, Fereidoun Azizi, Moein Yoosefi, Farshad Farzadfar, Davood Khalili

**Affiliations:** 1 Prevention of Metabolic Disorders Research Center, Research Institute for Endocrine Sciences, Shahid Beheshti University of Medical Sciences, No. 23, Parvaneh Street, Velenjak, Tehran, Iran; 2 Department of Biostatistics and Epidemiology, Research Institute for Endocrine Sciences, Shahid Beheshti University of Medical Sciences, Tehran, Iran; 3 Osteoporosis Research Center, Endocrinology and Metabolism Research Institute, Tehran University of Medical Sciences, Tehran, Iran; 4 Endocrine Research Center, Research Institute for Endocrine Sciences, Shahid Beheshti University of Medical Sciences, Tehran, Iran; 5 Non-Communicable Diseases Research Center, Endocrinology and Metabolism Population Sciences Institute, Tehran University of Medical Sciences, Tehran, Iran

**Keywords:** Cardiovascular diseases, Diabetes, Cardiovascular diseases, Disease prevention, Public health, Health care, Risk factors

## Abstract

To determine levels of change in risk factors for cardiovascular disease among people with and without a previous diagnosis of diabetes from 2007 to 2016 in Iran. Data were obtained from five rounds of the World Health Organization STEPwise approach to Surveillance (STEPS) cross-sectional surveys. Participants were 7665 and 93,733 adults with and without known diabetes, respectively, aged 25–65 years. We used logistic and linear regressions to assess the trends of risk factors. Individuals with known diabetes compared to those without the condition, experienced greater reductions in mean levels of systolic blood pressure (3.0 vs. 0.5 mmHg among women and 3.9 vs. 1.6 mmHg among men), diastolic blood pressure (6.4 vs. 5.11 mmHg in women and 3.3 vs. 1.8 mmHg in men), and non-HDL cholesterol (42.4 vs. 27.2 mg/dL among women and 30.3 vs. 21.0 mg/dL among men) throughout these years. Men with diabetes also showed a greater reduction in the prevalence of daily cigarette smoking compared to their non-diabetic counterparts (7.3% vs. 2.3%). Fasting plasma glucose decreased among subjects with diabetes but increased among those without diabetes. Significant increases were observed in proportions who met goals for blood pressure, triglycerides, non-HDL cholesterol and LDL cholesterol in both groups; however, almost half of diabetic subjects did not achieve risk factor goals in 2016. Secondary prevention in diabetic patients was more effective than primary prevention in the general population; however, the rate of diabetic patients who met the designated goals for each risk factor was still suboptimal.

## Introduction

Diabetes is associated with premature mortality from different causes, including cardiovascular diseases (CVD)^1^. Previous studies have demonstrated the vitality of CVD risk factor control to decrease the fast-rising trend of this mortality and CVD events in people with diabetes^2–4^. Furthermore, evidence suggests that the use of more intensive targets for blood pressure and cholesterol in individuals with diabetes results in a more significant reduction in the incidence of CVD events^5–7^. However, several studies report that people with diabetes are less likely to achieve target goals for control of CVD risk factors than those without diabetes^8,9^.

Nationally representative data that investigate trends in CVD risk factors in both the general population and people with diabetes in Middle Eastern countries such as Iran are scarce. Moreover, due to the lack of proper investigations that compare secular trends for CVD risk factors in individuals with and without diabetes in this region, it is difficult to illustrate the impact of preventative programs on this population and also prioritize strategies for primary and secondary preventions.

The primary goal of this analysis is to characterize and compare the trends of body mass index (BMI), waist circumference (WC), blood pressure, smoking, plasma glucose and lipid measures among adults with known diabetes (as indicators of secondary prevention impacts) and those without known diabetes (as indicators of primary prevention impacts) in a nationally representative study from 2007 through 2016.

## Results

Age and sex distribution of participants with and without known diabetes are presented in Table 1. The proportion of women was higher among those with known diabetes. The mean age for participants was approximately 44 years in all study years; however, for those with known diabetes, the mean age was almost ten years higher.

**Table 1:** Age and sex distribution of the participants with and without diagnosed diabetes in five STEPS surveys from 2007 to 2016.

	STEPS-2007	STEPS-2008	STEPS-2009	STEPS-2011	STEPS-2016
N	23,487	23,290	23,334	7551	23,738
Age, years	44.3 ± 11.4	44.2 ± 11.4	44.7 ± 11.5	44.4 ± 12.4	42.3 ± 11.1
Female, n (%)	11,683 (49.7)	11,539 (49.5)	11,545 (49.5)	4482 (59.4)	12,437 (52.4)
**Known diabetes**					
N, (%)	1608 (6.8)	1629 (7)	1571 (6.7)	787 (10.4)	2072 (8.7)
Age, years	52 ± 8.8	52.2 ± 8.6	52.8 ± 8.7	53.3 ± 9	50.7 ± 9.9
Female, n (%)	968 (60.2)	995 (61.1)	1003 (63.8)	518 (65.8)	1278 (61.7)
**No known diabetes**					
N, (%)	21,879 (93.2)	21,661 (93)	21,763 (93.3)	6764 (89.6)	21,666 (91.3)
Age, years	43.7 ± 11.3	43.7 ± 11.4	44.1 ± 11.4	43.4 ± 12.4	41.5 ± 10.9
Female, n (%)	10,715 (49)	10,544 (48.7)	10,542 (48.4)	3964 (58.6)	11,159 (51.5)

### Trends of risk factors in persons with known diabetes

**Figure 1:**
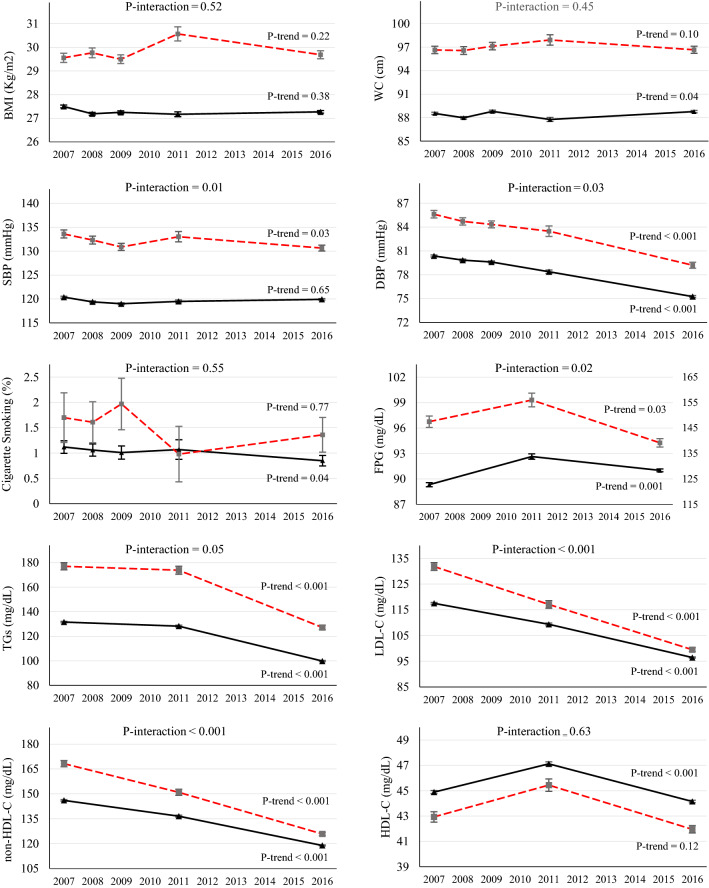
Trends of CVD risk factor levels among women with (red dotted line) and without diabetes (black dashed line) through 2007–2016. Changes in mean levels Body Mass Index (BMI), Waist Circumference (WC), Systolic Blood Pressure (SBP), Diastolic Blood Pressure (DBP) and Daily cigarette smoking are preented for five STEPS surveys in 2007, 2008, 2009, 2011 and 2016. Changes in Fasting Plasma Glucose FPG), Triglycerides (TGs), HDL cholesterol (HDL-C), non-HDL cholesterol (non-HDL-C) and LDL cholesterol throughout are presented for three STEPS surveys in 2007, 2011 and 2016.

**Figure 2:**
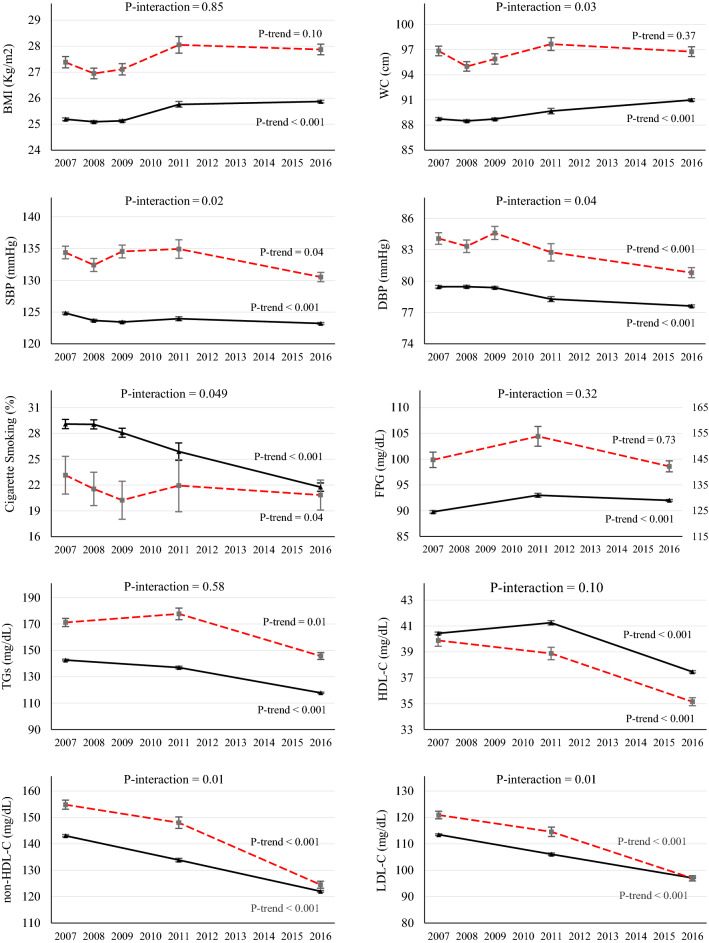
Trends of CVD risk factor levels among men with (red dotted line) and without diabetes (black dashed line) through 2007–2016. Changes in mean levels Body Mass Index (BMI), Waist Circumference (WC), Systolic Blood Pressure (SBP), Diastolic Blood Pressure (DBP) and Daily cigarette smoking are preented for five STEPS surveys in 2007, 2008, 2009, 2011 and 2016. Changes in Fasting Plasma Glucose FPG), Triglycerides (TGs), HDL cholesterol (HDL-C), non-HDL cholesterol (non-HDL-C) and LDL cholesterol throughout are presented for three STEPS surveys in 2007, 2011 and 2016.

Among those with diabetes, there were significant reductions of mean SBP (from 134 to 131 mmHg in women and 134 to 130 mmHg in men), DBP (from 86 to 79 mmHg in women and 84 to 81 mmHg in men), LDL cholesterol (from 132 to 99 mg/dL in women and 121 to 97 mg/dL in men), non-HDL cholesterol (from 168 to 126 mg/dL in women and 155 to 125 mg/dL in men) and triglycerides (from 177 to 127 mg/dL in women and 171 to 146 mg/dL in men) in both sexes. Furthermore, there were significant reductions in FPG among women (from 147 to 139 mg/dL) and HDL cholesterol among men (from 40 to 35 mg/dL) (Figs. 1 and 2). There was not enough evidence supporting a significant trend for BMI and waist circumference.

Table 2 shows the trends of meeting goals/optimum levels of risk factors among persons with and without known diabetes. In both sexes, there were significant increases in the proportion of participants with an optimum level of blood pressure (from 47 to 58% in women and 55 to 62% in men), LDL cholesterol (from 21 to 54% in women and 26 to 57% in men), non-HDL cholesterol (from 21 to 58% in women and 26 to 59% in men), and triglycerides (from 36 to 66% in women and 39 to 53% in men). Glycemic control improved in diabetic women (from 49 to 55%) (Table 2) but not in diabetic men. Nevertheless, the prevalence of subjects who met HDL cholesterol goal significantly reduced in both sexes (from 19 to 17% in women and 44 to 23% in men).

**Table 2:** Age-adjusted percentages of subjects with and without diagnosed diabetes who reached goals/optimum levels of blood pressure, fasting plasma glucose and lipid measures.

		STEPS-2007		STEPS-2011		STEPS-2016		P-trend	P-interaction
		n	% (SE)	n	% (SE)	n	% (SE)		
**Women**									
Blood pressure < 140/90 mmHg	KDM*	403	47.0 (1.9)	211	44.8 (2.7)	661	57.9 (1.6)	< 0.001	0.555
	Non-KDM	7424	74.6 (0.5)	2881	78.2 (0.7)	8867	84.1 (0.4)	< 0.001	
Fasting plasma glucose < 130 mg/dL	KDM	459	49.2 (1.8)	191	37.5 (2.4)	677	54.7 (1.5)	< 0.001	0.12
Fasting plasma glucose < 100 mg/dL	Non-KDM	8665	84.6 (0.4)	2934	76.9 (0.7)	8036	80.9 (0.4)	< 0.001	
HDL cholesterol > 50 mg/dL	KDM	200	19.4 (1.4)	136	24.7 (2.1)	218	16.8 (1.1)	0.032	0.532
	Non-KDM	2652	24.4 (0.5)	1297	31.4 (0.8)	2649	23.1 (0.4)	0.004	
Non-HDL cholesterol < 130 mg/dL	KDM	203	21.4 (1.5)	153	30.4 (2.3)	715	58.1 (1.5)	< 0.001	0.724
Non-HDL cholesterol < 160 mg/dL	Non-KDM	6890	67.6 (0.5)	2997	78.6 (0.7)	10,003	91.5 (0.3)	< 0.001	
LDL cholesterol < 100 mg/dL	KDM	205	21.2 (1.5)	153	29.3 (2.2)	678	54.5 (1.5)	< 0.001	0.798
LDL cholesterol < 130 mg/dL	Non-KDM	7057	69.1 (0.5)	3081	80.7 (.7)	10,048	91.8 (.3)	< 0.001	
Triglycerides < 150 mg/dL	KDM	349	36.3 (1.7)	179	35.9 (2.4)	807	65.7 (1.4)	< 0.001	0.011
	Non-KDM	7038	67.6 (0.5)	2682	70.1 (0.8)	9142	83.4 (0.4)	< 0.001	
**Men**									
Blood pressure < 140/90 mmHg	KDM	321	54.7 (2.4)	122	50.74 (3.6)	452	61.5 (2.0)	0.007	0.019
	Non-KDM	8138	76.7 (0.5)	2085	79.5 (0.9)	2338	83.6 (0.4)	< 0.001	
Fasting plasma glucose < 130 mg/dL	KDM	314	50.6 (2.2)	106	40.6 (3.4)	377	47.6 (2.0)	0.554	0.003
Fasting plasma glucose < 100 mg/dL	Non-KDM	9233	83.7 (0.4)	1952	74.3 (0.9)	8103	78.0 (0.4)	< 0.001	
HDL cholesterol > 40 mg/dL	KDM	290	43.6 (2.2)	116	38.8 (3.3)	199	23.2 (1.6)	< 0.001	0.189
	Non-KDM	5490	46.8 (0.5)	1557	54.6 (1.1)	3537	32.2 (0.5)	< 0.001	
Non-HDL cholesterol < 130 mg/dL	KDM	157	25.6 (2.0)	77	27.9 (3.0)	472	59.2 (2.0)	< 0.001	0.666
Non-HDL cholesterol < 160 mg/dL	Non-KDM	7768	71.0 (0.5)	2220	81.1 (0.8)	9429	90.7 (0.3)	< 0.001	
LDL cholesterol < 100 mg/dL	KDM	156	26.0 (2.0)	76	27.8 (3.0)	456	57.5 (2.0)	< 0.001	0.853
LDL cholesterol < 130 mg/dL	Non-KDM	8123	74.3 (0.5)	2319	84.5 (0.7)	9611	92.4 (0.3)	< 0.001	
Triglycerides < 150 mg/dL	KDM	244	38.7 (2.2)	91	32.0 (3.1)	428	53.4 (2.0)	0.015	0.966
	Non-KDM	6712	59.6 (0.5)	1764	63.2 (1.0)	7726	74.0 (0.5)	< 0.001	

*Known diabetes mellitus.

### Trends of risk factors among persons without known diabetes

There were a significant reduction in mean levels of lipid profiles, specially non-HDL cholesterol (from 146 to 119 mg/dL in women and 113 to 97 in men, 2007 vs. 2016) and triglycerides (from 132 to 100 mg/dL in women and 143 to 118 mg/dL in men) and prevalence of smoking (especially in men from 29.1 in 2007 to 21.8% in 2016) in both sexes. Regarding blood pressure, declining trends for mean DBP in both sexes and mean SBP in men were observed (Fig. 1). However, there was a slight, but statistically significant, increase in FPG (from 89 to 91 mg/dL in women and from 90 to 92 mg/dL in men, 2007 vs. 2016) and waist circumference (from 88.0 to 88.8 cm in women and from 88.7 to 91.0 in men). Only men had a significant increase in BMI (from 25.2 to 25.9 kg/m^2^) (Fig. 1).

In terms of optimal level of risk factors, we showed a significant increase in the proportion of those who had an optimal level of blood pressure (from 74.6 to 84.1% in women and 76.7 to 83.6% in men, 2007 vs. 2016), LDL cholesterol (from 69.1 to 91.8% in women and 74.3 to 92.4% in men), non-HDL cholesterol (from 67.6 to 91.5% in women and 71.0 to 90.7% in men), and triglycerides (from 67.6 to 83.4% in women and 59.6 to 74.0% in men) in both sexes. There were also decreasing trends in meeting optimal levels of FPG (from 84.6 to 80.9 in women and 83.7 to 78.0% in men) and HDL cholesterol (from 24.4 to 23.1% in women and 46.8 to 32.2% in men, 2007 vs. 2016) (Table 2).

### Difference in the trend of risk factors in persons with and without known diabetes

In both sexes, diabetic individuals showed a greater reduction of mean SBP (3.0 vs. 0.5 mmHg in women and 3.9 vs. 1.6 mmHg in men), DBP (6.4 vs. 5.11 mmHg in women and 3.3 vs. 1.8 mmHg in men), LDL cholesterol (32.4 vs. 21.1 mg/dL in women and 24.0 vs. 16.4 mg/dL in men), and non-HDL cholesterol (42.4 vs. 27.2 mg/dL in women and 30.3 vs. 21.0 mg/dL in men) compared to non-diabetics throughout 2007 to 2016 (Fig. 1).

Moreover, women without known diabetes showed an increase in FPG while there was a significant reduction among their counterparts. Waist circumference increased among men without known diabetes, while it remained unchanged among those with known diabetes (Fig. 1). Regarding meeting risk factor goals, women with diabetes had significantly more increase in the proportion that met goals of triglycerides compared to non-diabetic women (29.4 vs. 15.8). Additionally, compared to non-diabetic men, those with diabetes had significantly more reduction in the prevalence of smoking from 2007 to 2016 (8.1% vs. 2.3%) (Fig. 2).

## Discussion

In this study, we assessed national trends of cardio-metabolic risk factors from 2007 to 2016 in Iran concerning diabetes status. We provided some evidence on the effects of national efforts for primary and secondary prevention of cardio-metabolic disorders in a Middle Eastern country. There were significant improvements in blood pressure levels, non-HDL lipids, and daily smoking in the Iranian population; however, a deterioration in levels of HDL cholesterol was observed among those with and without diabetes. Importantly, we found increasing levels of obesity measurements and FPG in those without diabetes. Our findings suggest that secondary prevention has been more successful than primary prevention in reducing risk factors as they showed that changes in blood pressure, non-HDL cholesterol, FPG, and obesity (only in men) were more favorable in those with diabetes than those without the condition. Despite improvements over the past decade, meeting CVD risk factor goals, particularly among those with diabetes, remained suboptimal as almost half of Iranians with diabetes did not achieve risk factor goals.

Our findings regarding improvements in blood pressure and non-HDL lipid levels are in line with those of previous studies on Iranian populations, showing similar decreasing trends^10–12^ Notably, in this study, we had a higher proportion of participants, with and without diabetes who met goals of blood pressure and non-HDL lipids (53%-92%) compared with previous studies conducted in Iran (36%-87%)^10–12^. Our findings are also in line with those of the Global Burden of Disease (GBD) and NCD Risk Factor Collaboration (NCD-RisC), which showed reductions in the burden of high blood pressure and dyslipidemia in the Middle East and Worldwide^13–15^. Investigations from other countries have shown similar trends. Declines in mean SBP, DBP, LDL, non-HDL cholesterol and rate of smoking with more significant decreases among individuals with diabetes have been reported in studies from the National Health And Nutrition Examination Survey (NHANES) and the Framingham Heart Study in the US^16,17^ and the Health Survey for England ^18^. Accordingly, the prevalence of meeting blood pressure and LDL goals has been improved in the US^16,17^. Decreasing trends in high blood pressure were also found in studies from France and Japan, while the prevalence of hypercholesterolemia increased^19,20^. The results regarding HDL-cholesterol and triglycerides are different, from decreasing to no change or increasing^21,22^. Regarding the management of diabetes, in line with our findings, a study across 28 low-middle income countries uncovered poor control of diabetes, indicating a significant unmet need for diabetes care in these countries^23^. However, our results show that around 40–50% of our diabetic men and women reached the goal for glucose control, defined as FPG < 130, versus 23% on average in these 28 low-middle income countries, with a definition of FPG < 180 mg/dl; indicating much better situation of glucose control in Iran.

As one of the first countries from the Eastern Mediterranean Regional Office of WHO, Iran has responded to "a call for action" for the prevention and control of diabetes mellitus; and the national program was designed in 1996. The aim of this program is primary, secondary, and tertiary prevention, through community and high-risk screening, and the integration of diabetes care into the primary healthcare network. After the implementation in 2016 of IraPEN, an adaptation of WHO's Package of essential NCD (PEN) interventions for primary health care, this program integrated into the guidelines for prevention and control of major NCDs in the primary health care system. The current study shows that secondary prevention in diabetic patients has been more effective than primary prevention in the general population for controlling CVD risk factors, i.e., risk factors decreased more in diabetic patients than in non-diabetic subjects in the general population. However, the rate of diabetic patients who met the designated goals for each risk factor was still suboptimal.

The favorable changes may be rooted in nutritional habits, physical activity levels, smoking, and anti-hypertension or lipid-lowering drug consumption, all of which are known to be important determinants of blood pressure and serum lipid levels^10,11^. Previous studies showed that Iranian families had reduced the consumption of hydrogenated oil^24,25^ and salt^26^, which could explain the favorable blood pressure and lipid trends in Iranians. Further, previous studies showed increasing use of anti-hypertension or lipid-lowering drugs among Iranian^10–12^. Nevertheless, the decreasing trends in blood pressure and lipid levels in our population could hardly be explained by an increase in physical activity, since it was shown that low physical activity is common in Iranian community^27,28^.

Notably, by showing a significant reduction in the prevalence of daily smoking among those without known diabetes, this study suggests that efforts for lowering the prevalence of smoking have been successful in controlling the increasing trends^10^. The decrease in the prevalence of daily smoking in this group might also contribute to the decline in non-HDL lipid and blood pressure given the substantial evidence on the positive correlation of smoking with high blood pressure and dyslipidemia^29,30^.

On the contrary, we showed a rise in levels of obesity measurements in individuals without known diabetes (WC in both genders and BMI in men), which is probably the main reason for the increasing fasting plasma glucose trends in this group^31,32^. Contrary to the results of the previous studies that showed growing trends for HDL cholesterol in Iranian population^10^, this study showed a significant reduction in HDL cholesterol in those with and without diabetes. As shown in previous studies, decreasing levels of HDL cholesterol are mostly due to poor dietary habits and low levels of physical activity^33,34^. Deterioration in obesity measurements and HDL cholesterol levels should raise the alarm for policymakers as it indicates efforts for improvement of dietary habits and physical activity came short in this Iranian population. Based on previous studies in Iran^28,35–38^, the most important barriers to healthy nutrition and physical activity were interpersonal/cultural effects, lack of access to healthy foods, food preferences, media advertisements, nutrition transition, lack of time, motivation and prioritizing other activities over sports and high costs of the facilities. Therefore, feasible and effective national intervention programs are needed to curb current obesity epidemics by overcoming the barriers to healthy nutrition and physical activity. Despite the promising findings of several community-wide lifestyle intervention programs in Iran, such as Tehran Lipid and Glucose Study^39^ and Isfahan Healthy Heart Program^40^, these programs did not scale up in national levels.

In accordance with the evidence showing a correlation between diabetes and other cardio-metabolic risk factors, we showed risk factor levels were higher in those with diabetes compared to those without it^10,31^. Our findings support that the efforts on controlling blood pressure, dyslipidemia and obesity had more impacts among people with diabetes rather than non-diabetics. First of all, this is because diabetic patients experienced more reduction in mean SBP, DBP and non-HDL lipids. In addition, their obesity and blood glucose measurements remained steady, while people without diabetes showed a significant increase in obesity measurements. Favorable trends in dyslipidemia, obesity and blood glucose control in known diabetics may be due to improvements in care, knowledge and attitude towards diabetes as well as better adherence to lifestyle and pharmaceutical interventions. Previous studies show advancements in the quality of diabetes care, affordability of medications, and screening for undiagnosed diabetes as well as increasing trends in consumption of glucose-lowering (twofold in men and 1.5-fold in women) and lipid-lowering drugs (fourfold in men and 2.5-fold in women) among those with known diabetes^10,41^. Educational interventions in Iran, such as Self-Management Education (PDSME) program, also proved to be effective in improving the knowledge and practice of diabetes^42,43^, which is highly correlated with control of related risk factors^44^.

Nevertheless, in this study, the prevalence of daily smoking remained steady in participants with diabetes, while its prevalence reduced significantly in those without the disease. These findings are in contrast with those of a previous study that showed an increasing trend for smoking in those with diabetes was significantly higher than increases in smoking prevalence in those without diabetes^10^. This discrepancy may indicate favorable and radical changes in the prevalence of smoking. Another explanation may be the differences in the study sample and definition of smoking in this study compared to the previous studies, which were mostly cohort studies with local study samples and various definitions of smoking^10,11^. Further research is needed to provide more robust evidence on trends for smoking prevalence in Iran.

Since, during the last decades, the highest number of STEPS surveys in the Middle East have been conducted in Iran, it was made possible to investigate the secular trend of CVD risk factors using national data for the first time in this region^45^. Standardized measurement methods throughout the study period were used and large sample sizes and multiple measurements led to high precision in estimating the prevalence and trends of different risk factors. However, there are some limitations. We only assessed these trends for 10 years. Although the sampling methods were representative of the Iranian population, there were some minor differences that were addressed using post-stratification weighting based on age, sex, region and province categories of population in 2011 as a reference for all years. Finally, the biochemical measurements were only available for three STEPs cycles.

## Conclusion

Non-HDL lipids and blood pressure levels in the Iranian population improved significantly in those with and without diabetes. Moreover, obesity measurements and fasting plasma glucose worsened in diabetic persons. This study showed favorable changes in blood pressure, non-HDL cholesterol, blood glucose, and obesity (only in men) were more prominent in people with diabetes compared to those without known diabetes. This indicating secondary prevention efforts have been more effective than primary prevention in Iran.

## Methods

This study is conducted on the data documented from five STEPwise approaches to Surveillance (STEPS) surveys (2007, 2008, 2009, 2011, and 2016). The first STEPS in 2005 was not included due to the inconsistency of some measurement methods. STEPS is a standardized survey designed to help the World Health Organization (WHO) member states collect and disseminate consistent data about non-communicable diseases (NCD) and enable comparisons over time. A brief explanation of the samplings of the surveys is presented as follows.

All methods in the current study were carried out following relevant guidelines and regulations, the Center for Disease Control, Ministry of Health and Medical Education in Iran approved all experimental protocols, and all participants gave informed consent.

### Study population

Using random cluster sampling methods based on instructions of WHO for STEPS^45^ 23,487, 23,290, 23,334, 7551, and 23,738 adults, aged 25–65, were selected in 2007, 2008, 2009, 2011 and 2016, respectively. Despite differences in design and sampling methods, all surveys represented the Iranian population. Socio-demographic information and physical measurements were collected in all five surveys, whereas biochemical measurements were collected only in 2007, 2011 and 2016.

The sampling method in 2007, 2008 and 2009 was similar. The sampling was conducted at levels of towns, villages and districts of large cities using a randomized cluster sampling scheme. In each province, 1000 individuals were selected in 50 clusters. Each cluster included 20 individuals, 10 women and 10 men, living in neighboring households.

The 2011 survey used a multistage cluster random sampling scheme. At the first stage, distinct counties or a group of adjacent counties were listed as the primary sampling units (PSU). Fifty PSUs were then picked by applying probability proportionate to size (PPS) random sampling. Within each PSU, urban and rural areas were listed as possible secondary sampling units (SSU) from which twelve SSUs were picked by employing the PPS method similar to the previous step. In the third stage, a list of households in each SSU referenced by their 10-digit postal codes was developed, of which twenty postal addresses were selected randomly. At the final stage, one individual was selected from each selected household using Kish tables provided by WHO, and they were interviewed at their houses.

In 2016, a systematic cluster random sampling scheme was designed to select 31,050 individuals in 3105 clusters (10 subjects from each cluster) from urban and rural areas of all provinces. To estimate the minimum sample size at the province level, calculations were based on the province with the lowest population density. The sample size in other provinces was determined according to their population ratio to that province. To control non–response errors and mind the effect of sampling design, 10% was added to the estimated sample size of each province. Also, to minimize costs, in more crowded provinces (those with more than 800 clusters), half of the estimated sample size was considered, but the applied weight in the subsequent analysis was doubled.

### Medical history, clinical examination and laboratory measurements

Standard questionnaires based on the WHO STEPS were used to collect demographic information as well as the past medical history of diabetes, medication use and cigarette smoking. Blood pressure was measured three times at five-minute intervals. The average of second and third readings was considered as the participants' blood pressure.

All biochemical measurements were assessed in venous samples drawn after 12–14 h overnight fasting according to a standard protocol and sent to collaborating centers. In 2007 and 2011, Fasting Plasma Glucose (FPG) was measured with enzymatic colorimetric methods with a glucose oxidase test and serum lipids, including total cholesterol, high-density lipoprotein (HDL) cholesterol and triglycerides were determined by enzymatic methods (Pars Azmun, Karaj, Iran). In 2016, venous samples were transferred to the Central Reference Laboratory in Tehran and all laboratory tests were measured using the auto analyzer (Cobas C311 Hitachi High–Technologies Corporation) approved by Reference Laboratory. Low-density lipoprotein (LDL) cholesterol was calculated by the modified Friedewald equation to be consistent among all STEPS surveys^46^.

### Definition of terms

Known diabetes was defined as a positive response to either of the two following questions: (1) “Have you ever been told by a doctor or other health worker that you have diabetes?” and (2) “Are you currently taking insulin or oral medication for diabetes prescribed by a doctor or other health worker?”. Hypertension goal/optimum level was defined as mean systolic blood pressure (SBP) < 140 mmHg or mean diastolic blood pressure (DBP) < 90 mmHg^47^. Participants who smoked cigarettes daily were considered 'daily smokers'. Glycemic control was defined as a goal of FPG < 130 mg/dL in diabetic subjects and an optimum level of FPG < 100 mg/dL in non-diabetic subjects. Goals/optimum levels for non-HDL cholesterol and LDL cholesterol were defined as non-HDL cholesterol < 130 mg/dl and LDL cholesterol < 100 mg/dl in those with known diabetes and non-HDL cholesterol < 160 mg/dl and LDL cholesterol < 130 mg/dL in those without known diabetes. Goals/optimum levels for HDL cholesterol were defined as ≥ 50 mg/dl for women and ≥ 40 mg/dl for men and for triglycerides were defined as triglyceride < 150 mg/dl in both genders^47,48^.

### Statistical analysis

Characteristics of the study population were described by mean (SE) values for continuous variables and frequency (%) for categorical variables after accounting for the survey nature of the data. Since about 10% of the data were missed, the multiple imputation method was used for the estimation of missing information. Five imputation sets were performed to impute missing information of FPG, TG, total cholesterol, BMI, and blood pressure, using sex, area of living, and age as axillary variables. Survey analysis was performed in Stata ver. 12 and the results of all STEPS were weighted using "poststratum weights" according to the national Iranian census in 2011; "poststrata" was defined based on categories of age (25–44 years, 45–64 years), sex, residential area (rural, urban) and the provinces; in this way, the results of all STEPS would be comparable.

The trend of prevalence rates and averages of risk factors in diabetic and non-diabetic subjects were examined separately using logistic and linear regressions. The interaction between the trend of risk factors and diabetes status was evaluated by adding the interaction term of time × diabetes to the models in pooled data from diabetic and non-diabetic subjects.

## Supplementary information

Supplementary file1
