# Supplementary material for: Trends in cardiovascular risk factors in diabetic patients in comparison to general population in Iran: findings from National Surveys 2007–2016
Source: Sci Rep. 2020 Jul 16;10:11724. doi: 10.1038/s41598-020-68640-9 (PMC7366682; doi:10.1038/s41598-020-68640-9)
Supplement: Supplementary file 1 — Supplementary file1 [file 41598_2020_68640_MOESM1_ESM.docx]

**Full title:**

Trends in Cardiovascular Risk Factors in Diabetic Patients in Comparison to General Population in Iran: Findings from National Surveys 2007-2016

**Authors:**

Hamid Malekzadeh^1^, Mojtaba Lotfaliany^1, 2^, Afshin Ostovar^3^, Farzad Hadaegh^1^, Fereidoun Azizi^4^, Moein Yoosefi^5^, Farshad Farzadfar^5^, Davood Khalili^1, 2^*

**Affiliations:**

^1^ Prevention of Metabolic Disorders Research Center, Research Institute for Endocrine Sciences, Shahid Beheshti University of Medical Sciences, Tehran, Iran.

^2^ Department of Biostatistics and Epidemiology, Research Institute for Endocrine Sciences, Shahid Beheshti University of Medical Sciences, Tehran, Iran.

^3^ Osteoporosis Research Center, Endocrinology and Metabolism Research Institute, Tehran University of Medical Sciences, Tehran, Iran

^4^ Endocrine Research Center, Research Institute for Endocrine Sciences, Shahid Beheshti University of Medical Sciences, Tehran, Iran.

^5^ Non-Communicable Diseases Research Center, Endocrinology and Metabolism Population Sciences Institute, Tehran University of Medical Sciences, Tehran, Iran

* **Correspondence to:**

Davood Khalili, MD, MPH, PhD

Associate Professor of Epidemiology,

Prevention of Metabolic Disorders Research Center & Head of Department of Biostatistics and Epidemiology, Research Institute for Endocrine Sciences, Shahid Beheshti University of Medical Sciences, Tehran, Iran

Postal Address: No. 23, Parvaneh Street, Velenjak, Tehran, Iran

Phone: +98-21- 22432500

Fax: +98-21- 22416264

E-mail address: [dkhalili@endocrine.ac.ir](mailto:dkhalili@endocrine.ac.ir)

| Supplementary table 1. Average measures of BMI, WC, SBP and DBP in subjects with and without diabetic in STEPS Study (2007-2016). | | | | | | | | | | | | |
| --- | --- | --- | --- | --- | --- | --- | --- | --- | --- | --- | --- | --- |
|  | | STEPS-2007 | | STEPS-2008 | | STEPS-2009 | | STEPS-2011 | | STEPS-2016 | | P-trend |
| **Women** | |  |  |  |  |  |  |  |  |  |  |  |
| BMI* (kg/m^2^) (Mean (SE)) | KDM**^‡^** | 29.56 (0.20) | | 29.77 (0.21) | | 29.5 (0.18) | | 30.57 (0.31) | | 29.69 (0.17) | | 0.218 |
|  | non-KDM | 27.5 (0.06) | | 27.19 (0.06) | | 27.25 (0.06) | | 27.17 (0.10) | | 27.27 (0.06) | | 0.380 |
| WC**^§^** (cm) (Mean (SE)) | KDM | 96.64 (0.47) | | 96.57 (0.50) | | 97.13 (0.48) | | 97.92 (0.68) | | 96.66 (0.47) | | 0.104 |
|  | non-KDM | 88.54 (0.15) | | 87.98 (0.16) | | 88.79 (0.17) | | 87.77 (0.25) | | 88.77 (0.15) | | 0.038 |
| Systolic blood pressure (mmHg) (Mean (SE)) | KDM | 133.61 (0.84) | | 132.33 (0.84) | | 130.90 (0.76) | | 133.03 (1.09) | | 130.65 (0.63) | | 0.026 |
|  | non-KDM | 120.38 (0.21) | | 119.40 (0.2) | | 119.00 (0.2) | | 119.50 (0.31) | | 119.93 (0.17) | | 0.650 |
| Diastolic blood pressure (mmHg) (Mean (SE)) | KDM | 85.62 (0.47) | | 84.72 (0.47) | | 84.34 (0.43) | | 83.48 (0.67) | | 79.20 (0.37) | | <0.001 |
|  | non-KDM | 80.37 (0.13) | | 79.84 (0.14) | | 79.61 (0.13) | | 78.38 (0.21) | | 75.26 (0.12) | | <0.001 |
| **Men** |  |  |  |  |  |  |  |  |  |  |  |  |
| BMI (kg/m^2^) (Mean (SE)) | KDM | 27.38 (0.22) | | 26.95 (0.21) | | 27.11 (0.22) | | 28.05 (0.32) | | 27.87 (0.21) | | 0.097 |
|  | non-KDM | 25.19 (0.05) | | 25.09 (0.05) | | 25.13 (0.05) | | 25.76 (0.11) | | 25.87 (0.05) | | <0.001 |
| WC (cm) (Mean (SE)) | KDM | 96.84 (0.59) | | 94.99 (0.59) | | 95.88 (0.63) | | 97.66 (0.78) | | 96.75 (0.61) | | 0.373 |
|  | non-KDM | 88.73 (0.15) | | 88.49 (0.15) | | 88.71 (0.14) | | 89.66 (0.33) | | 90.99 (0.15) | | <0.001 |
| SBP (mmHg) (Mean (SE)) | KDM | 134.37 (1.01) | | 132.40 (1.06) | | 134.53 (1.01) | | 134.92 (1.46) | | 130.51 (0.76) | | 0.037 |
|  | non-KDM | 124.84 (0.17) | | 123.68 (0.18) | | 123.44 (0.17) | | 123.96 (0.32) | | 123.21 (0.15) | | <0.001 |
| DBP (mmHg) (Mean (SE)) | KDM | 84.08 (0.56) | | 83.34 (0.62) | | 84.61 (0.66) | | 82.76 (0.83) | | 80.81 (0.50) | | <0.001 |
|  | non-KDM | 79.46 (0.13) | | 79.47 (0.13) | | 79.38 (0.12) | | 78.28 (0.25) | | 77.62(0.11) | | <0.001 |
| * Body Mass Index  **^‡^** Known Diabetes Mellitus  **^§^** Waist Circumference | | | | | | | | | | | | |

| Supplementary table 2. Average measures of FBS and lipid measures in subjects with and without diabetic in STEPS Study (2007-2016). | | | | | |
| --- | --- | --- | --- | --- | --- |
|  | | n (%) | | |  |
|  | | STEPS-2007 | STEPS-2011 | STEPS-2016 | P-trend |
| **Women** | |  |  |  |  |
| Fasting plasma glucose (mgr/dL) (Mean (SE)) | KDM* | 147.40 (2.26) | 155.35 (2.63) | 139.30 (1.65) | 0.025 |
|  | non-KDM | 89.26 (0.23) | 92.64 (0.33) | 91.00 (0.15) | 0.001 |
| HDL cholesterol (mgr /dL) (Mean (SE)) | KDM | 42.93 (0.41) | 45.44 (0.49) | 41.95 (0.29) | 0.122 |
|  | non-KDM | 44.89 (0.12) | 47.12 (0.15) | 44.15 (0.10) | <0.001 |
| Triglycerides (mgrl/dL) (median (IQR)) | KDM | 177 (2.98) | 173.83 (3.30) | 127.16 (1.89) | <0.001 |
|  | non-KDM | 131.58 (0.57) | 128.27 (0.82) | 99.81 (0.45) | <0.001 |
| Non-HDL cholesterol (mgr/dL) (Mean (SE)) | KDM | 168.23 (1.88) | 150.95 (1.84) | 125.82 (1.05) | <0.001 |
|  | non-KDM | 146.07 (0.44) | 136.54 (0.63) | 118.89 (0.31) | <0.001 |
| LDL cholesterol (mgr/dL) (Mean (SE)) | KDM | 131.85 (1.53) | 117.09 (1.48) | 99.50 (0.78) | <0.001 |
|  | non-KDM | 117.53 (0.36) | 109.37 (0.51) | 96.45 (0.24) | <0.001 |
| **Men** |  |  |  |  |  |
| Fasting plasma glucose (mgr/dL) (Mean (SE)) | KDM | 144.55 (3.10) | 153.38 (3.77) | 141.95 (2.08) | 0.730 |
|  | non-KDM | 89.69 (0.25) | 93.05 (0.37) | 92.06 (0.19) | <0.001 |
| HDL cholesterol (mgr/dL) (Mean (SE)) | KDM | 39.88 (0.44) | 38.88 (0.47) | 35.15 (0.32) | <0.001 |
|  | non-KDM | 40.43 (0.10) | 41.25 (0.16) | 37.46 (0.09) | <0.001 |
| Triglycerides (mgr/dL) (median (IQR)) | KDM | 171.16 (3.15) | 177.63 (4.50) | 145.67 (2.66) | 0.008 |
|  | non-KDM | 142.63 (0.65) | 136.96 (1.04) | 117.84 (0.57) | <0.001 |
| Non-HDL cholesterol (mgr/dL) (Mean (SE)) | KDM | 154.82 (1.73) | 148.01 (2.17) | 124.52 (1.34) | <0.001 |
|  | non-KDM | 143.07 (0.41) | 133.87 (0.65) | 122.04 (0.31) | <0.001 |
| LDL cholesterol (mgr/dL) (Mean (SE)) | KDM | 120.91 (1.42) | 114.55 (1.77) | 96.94 (0.99) | <0.001 |
|  | non-KDM | 113.48 (0.33) | 106.07 (0.51) | 97.11 (0.24) | <0.001 |
| * Known Diabetes Mellitus | | | | | |
